# Therapeutic strategies to overcome EGFR mutations as acquired resistance mechanism in ALK-rearranged non-small-cell lung cancer: Case Reports

**DOI:** 10.3389/fonc.2023.1182558

**Published:** 2023-06-28

**Authors:** Lionel Michaux, Alexandre Perrier, Camille Mehlman, Hussa Alshehhi, Antonin Dubois, Roger Lacave, Florence Coulet, Jacques Cadranel, Vincent Fallet

**Affiliations:** ^1^ Department of Pulmonology and Thoracic Oncology, Assistance Publique Hôpitaux de Paris, Hôpital Tenon and Groupe de Recherche Clinique 4 (GRC 4), Theranoscan, Sorbonne Université, Paris, France; ^2^ Genetics Department, Assistance Publique Hôpitaux de Paris, Hôpital de la Pitié-Salpêtrière and Sorbonne Université, Paris, France; ^3^ Pathology Department, Assistance Publique Hôpitaux de Paris, Hôpital Tenon and Sorbonne Université, Paris, France; ^4^ Department of Pharmacy, Assistance Publique Hôpitaux de Paris, Hôpital Tenon and Sorbonne Université, Paris, France

**Keywords:** non-small cell lung cancer, ALK rearrangement, tyrosine kinase inhibitors, resistance mutation, EGFR

## Abstract

**Introduction:**

ALK tyrosine kinase inhibitors (ALK TKIs) have improved prognosis in *ALK*-rearranged (*ALK* ^+^) non-small-cell lung cancer (NSCLC). However, drug resistance mechanisms occur inevitably during the course of treatment leading to disease progression. Activation of epidermal growth factor receptor (EGFR) bypass signaling pathway is an uncommon cause of acquired resistance to ALK TKIs.

**Method:**

We present two patients with *EML4-ALK* rearranged NSCLC, developing an acquired *EGFR* resistance mutation after receiving multiple lines of ALK TKIs.

**Results:**

While preclinical models have showed encouraging data, there is a critical need for clinical studies on treatment strategies to overcome this drug resistance. Three real-life therapeutic approaches were used in this report: i) using brigatinib, an inhibitor targeting both ALK and EGFR tyrosine kinases; ii) combining two ALK TKIs together; and iii) delivering doublet platinum chemotherapy. In case 1, time to treatment failure (TTF) was 9.5 months with brigatinib; in case 2, TTF was 10 months with combined TKIs (osimertinib and brigatinib), whereas TTF with chemotherapy was only 2 months. Tolerability profile TKIs combotherapy was acceptable.

**Conclusion:**

These case reports underline the therapeutic complexity of *EGFR*-acquired resistance mutation in *ALK^+^* NSCLC and offers some leads to solve this real-life clinical challenge.

## Introduction

Giving an ALK tyrosine kinase inhibitor (ALK TKI) is the standard of care for first-line treatment of patients with advanced *ALK* rearrangement driven non-small-cell lung cancer (*ALK+* NSCLC).

However, nearly all patients develop acquired resistance to ALK TKIs. Resistance can be divided into two categories: in-target and off-target resistance mechanisms. Among these, the activation of alternative bypass signaling pathways have been described. We report two cases of rare resistance mechanisms involving EGFR pathway occurring after sequential treatment by ALK TKIs. We also aimed to describe our therapeutic approaches to overcome these EGFR-driven resistances.

## Cases presentation

### Case 1

**Figure 1:**
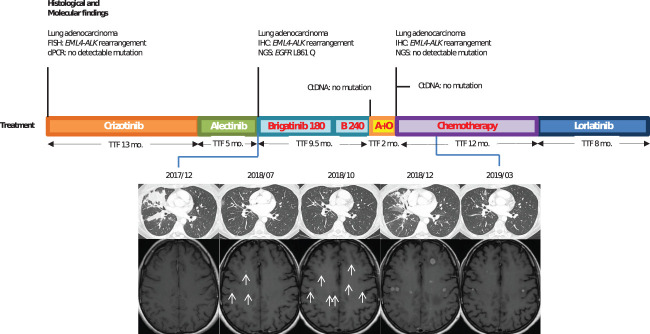
Case 1: timeline illustrating the changes in therapeutic regimen in correlation with molecular and radiological findings. FISH, fluorescence *in situ* hybridization; dPCR, digital polymerase chain reaction; TTF, time to treatment failure; Mo., months; ctDNA, circulating tumor DNA; IHC, immunohistochemestry; NGS, next-generation sequencing; Brigatinib 180, brigatinib with a 180-mg daily dose regimen; B240, brigatinib with a 240-mg daily dose regimen; A+O, combined treatment with alectinib and osimertinib. In red, the treatments used when *EGFR* resistance mutation was identified.

A then 38-year-old, non-smoker woman, presented to the hospital with chronic cough and asthenia in June 2016. Computed tomography (CT) scan showed a middle lobe consolidation with infradiaphragmatic lymphatic nodes. The patient was subsequently diagnosed with a metastatic lung adenocarcinoma. ALK immunohistochemistry (IHC) was positive with a strong intensity score (clone D5F3), and fluorescence *in situ* hybridization (FISH) confirmed an *EML4-ALK* fusion (Vysis LSI ALK Break Apart FISH Probe Kit from Abbott Molecular^®^). No additional somatic gene alteration, especially *EGFR* mutation, was found (EGFR genotyping was done using RT-PCR on QX200 system, BIO RAD^®^ system and TaqMan^®^ probes from Life Technologies^®^ for exons 18, 20, and 21 and fragment analysis by capillary gel electrophoresis on 3130 ABI system™ from Applied Technologies^®^ for exon 19). As first-line treatment, the patient received crizotinib (250 mg twice daily) for 13 months until brain magnetic resonance imaging (MRI) revealed brain metastases. After 5 months of treatment with alectinib (600 mg twice daily), CT scan showed progression of lung lesions (**Figure 1**). Next-generation sequencing (NGS) of tumoral lung tissue DNA revealed an *EGFR* L861Q mutation (c.2582T>A, p.(Leu861Gln))—variant allelic frequency (VAF) of 1.7% (Tumor Hotspot MASTR™ Plus, Multiplicom^®^). While *EML4-ALK* rearrangement was still found, no ALK-dependent resistance mutation was identified. Alectinib was thus discontinued, and brigatinib (90 mg once daily for 1 week, then 180 mg once daily) was given to the patient. The CT scan showed reduction in lung opacities after 8 weeks. Nevertheless, a differential response was observed after 6 months of treatment with maintained lung response but a progression of brain lesions on MRI. Brigatinib was then increased to 240 mg (once daily), and with this dose regimen, the patient had grade 1 myalgia and increased levels of blood creatinine phosphokinases, aspartate aminotransferases, and lipases. After 3 months with brigatinib increased dose, brain metastases progressed, and brigatinib was discontinued. No mutation was shown on plasma circulating tumor DNA, and the patient received a combination of alectinib 600 mg (twice daily) and osimertinib 80 mg (once daily) as fourth-line therapy, which was discontinued after 2 months because of progression. Seven additional lines of treatment were given to the patient, including carboplatin pemetrexed doublet chemotherapy (fifth line) and lorlatinib (sixth line), with 12 and 8 months of response to treatment, respectively. ALK resistance compound mutations (*ALK G1202R* and *ALK G1269A*) were first detected after lorlatinib treatment and were still identified until the patient’s death 74 months after the diagnosis.

### Case 2

**Figure 2:**
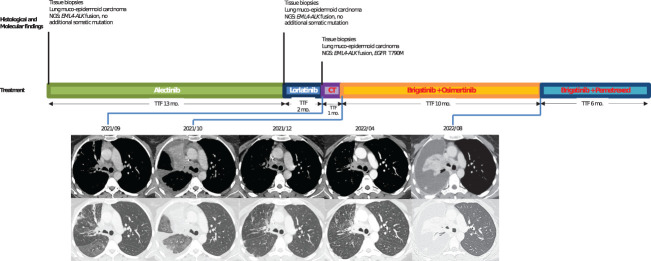
Case 2: timeline illustrating the changes in therapeutic regimen in correlation with molecular and radiological findings. NGS, next-generation sequencing; TTF, time to treatment failure; Mo., months; CT, chemotherapy. In red, the treatments used when *EGFR* resistance mutation was identified.

In June 2020, a 42-year-old, non-smoker woman was admitted to the hospital for chronic cough and dyspnea. Imaging tests showed a right upper lobe lung mass with secondary bones and liver lesions. CT-guided liver biopsies revealed a muco-epidermoid lung carcinoma. An immunohistological analysis revealed that TTF1 was negative, while the tumor cells without mucus were positive for p40. Tumor cells were also strongly positive for ALK IHC (clone D5F3). *EML4-ALK* fusion variant 1 was detected by RNA sequencing (Archer^®^ FusionPlex™ Lung panel). No additional somatic gene alteration (such as TP53, KRAS, and KEAP1) was shown in the NGS DNA analysis (SOPHIA Solid Tumor Solution™, SOPHIA Genetics^®^). Alectinib 600 mg twice daily was started, and the patient experienced a partial response for 12 months. When imaging revealed progression with pelvic bone metastases, lorlatinib was initiated with a 100-mg daily dose regimen. After 2 months, the CT scan revealed new lung opacities that were attributed to a lymphangitic carcinomatosis (**Figure 2**). Lorlatinib was discontinued, and a carboplatin–paclitaxel doublet chemotherapy was started but was rapidly stopped because of radiological evidences of lung progression. Meanwhile, DNA-based NGS (AmpliSeq™ for Illumina^®^ Focus Panel) in the tissue specimen from bronchus biopsies performed during progression under lorlatinib revealed an exon 20 *EGFR* T790M mutation (VAF, 1.3%) and the RNA sequencing the *EML4-ALK* rearrangement. The patient then started on a combination of brigatinib (90 mg once daily for 1 week, then 180 mg once daily) and osimertinib (80 mg once daily). Partial response in the lung was achieved after 6 weeks. The patient experienced, as treatment emergent adverse events, grade 2 vomiting and grade 1 thrombopenia. The patient remained on these combined therapies for 10 months until the CT scan showed pleural progression. As additional line of treatment, brigatinib and pemetrexed were given to the patient with a response of 6 months. The patient finally died 33 months after the diagnosis.

## Discussion

We reported here two cases of well-documented *EGFR* mutations linked to acquired resistance to ALK TKIs in *ALK*+ NSCLC. To our knowledge, neither L861Q nor T790M *EGFR* mutants have been described yet as EGFR bypass ALK resistance mechanism. These *EGFR* mutations were not detected at diagnosis even using different highly sensitive detection methods, making the possibility of *de novo* co-alterations unlikely.

Most frequently, *ALK+* NSCLCs are adenocarcinomas, but in case 2, it was a very rare subtype of NSCLC: mucoepidermoid carcinoma. *EML4-ALK* rearrangements in this rare subtype have been described in small series, including a case report with durable response to alectinib (1, 2).

Initially described in preclinical studies, the activation of EGFR signaling was identified as an infrequent post-ALK TKIs bypass resistance mechanism (3). In the largest clinical cohort that evaluated the mechanisms of resistance post-ALK TKIs, the most frequent alteration reported was the acquisition of an *ALK* resistance mutation in 56% of biopsies post second-generation ALK inhibitors, and no *EGFR* acquired resistance was identified is this cohort (4). In smaller cohorts, *EGFR* mutation as a mechanism of resistance to ALK TKIs has been previously shown (5–7). In the four described patients, activating *EGFR* mutations (L858R or exon 19 deletion) were found in tissue biopsies.

Therapeutic strategy proposed in this context has not been clarified. In the present report, we experienced three clinical therapeutic strategies trying to overcome the activation of EGFR signaling pathway. As a first strategy, we switched alectinib for brigatinib, which was of interest because of brigatinib dual ALK and EGFR activity (8). In cellular assays, brigatinib has been reported to have a substantial activity against *EGFR* exon 19 deletion, with potency only sevenfold reduced compared to *ALK (* 8). Moreover, preclinical (9) and clinical (10) data demonstrated that a combined targeted therapy of brigatinib and cetuximab could be beneficial to overcome the triple *EGFR* mutant (*EGFR T790M* and *cis-C797S*) resistance to osimertinib. However, brigatinib exhibits more modest activity against *EGFR L858R* or variants with *T790M* mutation (8), and there is no preclinical data supporting such an activity in case of rare *L861Q EGFR* mutation harbored by our patient. This strategy of using brigatinib to overcome *EGFR*-acquired resistance raises some limit in our first case with EGFR co-alteration. Even if no *ALK* resistance mutation was detected with the *EGFR* mutation after alectinib, the impact of brigatinib on this bypass resistance mutation remains uncertain, since this TKI also overcomes *ALK*-dependent resistance post-alectinib (11, 12). Finally, because of the isolated and asymptomatic brain progression, we chose to escalate brigatinib dose regimen to 240 mg daily. Despite a good tolerance, this strategy appeared to be ineffective. In the Phase 2 ALTA-2 trial, 13 patients escalated their brigatinib dosage from 180 to 240 mg daily after experiencing disease progression. While this higher dosage demonstrated an acceptable safety profile, the clinical benefit was disappointing, as no confirmed tumoral response was observed, and the progression-free survival was <2 months (13).

As an alternative therapeutic strategy, we experienced a combination treatment with both ALK and EGFR TKIs (alectinib + osimertinib in case 1 and brigatinib + osimertinib in case 2). No grade 3 toxicity was observed in both cases. An encouraging response was reported in case 2 in the fourth line after a rapid progression with both lorlatinib then chemotherapy. Concurrent inhibition of both EGFR and ALK is therapeutically effective in all of the ALK-resistant preclinical models, which have acquired resistance through EGFR pathway activation (3, 14) and has been described to be safe in several case reports in EGFR-resistant lung cancer with acquired ALK fusion co-alteration (15). However, in case 1, patient progressed dramatically after 2 months of combined therapies perhaps because the tumor was no longer EGFR dependent at this time.

Finally, a platinum-based doublet chemotherapy might be beneficial after failure of second- or third-generation ALK TKI in *ALK* rearranged NSCLC (16). Interestingly, our second patient rapidly progressed after 1 month of chemotherapy, while a prolonged (12 months) partial response occurred in the first patient. Nevertheless, this last result must be interpreted with caution given that the *EGFR* mutation was not present after treatment with brigatinib.

In conclusion, we reported two cases of *EGFR* mutant emerging after at least two lines of ALK TKIs in *ALK*-rearranged NSCLC. In order to overcome drug resistance, we adopted different strategies with variable efficacy. These case reports highlight both the complexity of drug resistance mechanisms and the therapeutic challenges in developing strategies to overcome drug resistance in oncogenic-driven NSCLC.

## Data availability statement

The raw data supporting the conclusions of this article will be made available by the authors, without undue reservation.

## Ethics statement

Ethical review and approval was not required for the study on human participants in accordance with the local legislation and institutional requirements. The patients/participants provided their written informed consent to participate in this study. Written informed consent was obtained from the individual(s) for the publication of any potentially identifiable images or data included in this article.

## Author contributions

LM: Conceptualization, Methodology, Investigation, Writing – Original draft. AP: Writing – Reviewing and Editing. CM: Writing – Reviewing and Editing. HA: Writing – Reviewing and Editing. AD: Writing – Reviewing and Editing. RL: Writing – Reviewing and Editing. FC: Writing – Reviewing and Editing. JC: Writing – Reviewing and Editing, Supervision. VF: Conceptualization, Methodology, Investigation, Writing – Original draft. All authors contributed to the article and approved the submitted version.
